# Immunomodulatory activity of glycoproteins isolated from chickpea (*Cicer arietinum* L.)

**DOI:** 10.3389/fnut.2022.966705

**Published:** 2022-09-16

**Authors:** Zhenxing Shi, Shiyu Li, Zuchen Wei, Yuanji Wang, Nong Zhou, Qiang Ma, Yang Yao

**Affiliations:** ^1^Institute of Crop Science, Chinese Academy of Agricultural Sciences, Beijing, China; ^2^Department of Basic Medicine, Chongqing Three Gorges Medical College, Chongqing, China; ^3^School of Food Science and Technology, Henan University of Technology, Zhengzhou, China; ^4^Laboratory for Green Cultivation and Deep Processing of Three Gorges Reservoir Area's Medicinal Herbs, College of Life Science and Engineering, Chongqing Three Gorges University, Chongqing, China

**Keywords:** glycoprotein, structural characterization, immunomodulatory, RAW 264.7 murine macrophage cell, chickpea

## Abstract

Chickpea (*Cicer arietinum* L.) is a well-known legume widely used as traditional medicine. This study aimed to characterize the structure and evaluate the immunomodulatory activity of one glycoprotein [crude chickpea glycoprotein-1 (CAG-1)] isolated from chickpea. CAG-1 was extracted with hot alkaline water and purified with DEAE-Sepharose Fast Flow and Superdex-200 column chromatography. CAG-1, with a molecular weight of 8,106 Da, contained 57.12% polysaccharide and 35.41% protein. The polysaccharide part was mainly composed of glucose (Glc). The protein part was connected mainly by aspartic (Asp) and glutamic (Glu). The results of nuclear magnetic resonance (NMR) analysis indicated the presence of α-d-Glc*p*-(1 → 4)-α-d-Glc*p*-(1 → 4)-α-d-Glc*p*-(1 → . In addition, the sugar chains of the glycoprotein were not hydrolyzed under alkaline conditions, suggesting that the glycoprotein was N-glycosidic; thus, the sugar chain was linked to the protein chain by Asp. An immunological study showed that CAG-1 stimulated the production of nitric oxide (NO), interleukin-6 (IL-6), tumor necrosis factor-α (TNF-α), and monocyte chemotactic protein 1 (MCP-1) in RAW 264.7 macrophages in a dose-dependent manner.

## Introduction

An innate immune system is the body's first line of defense against harmful foreign substances, such as bacteria, viruses, and fungi (1). Macrophages are important effector cells of the immune system. Activation of macrophages to release molecules with immunomodulatory activity, such as nitric oxide (NO), interleukin-1β (IL-1β), and tumor necrosis factor-α (TNF-α), has been reported to play a key role in the resistance to external pathogens (2). In recent years, various natural products from plants were demonstrated to exhibit immunomodulatory activity through the stimulation of the release of proinflammatory cytokines, with low toxicity and side effects (3, 4).

Glycoprotein is composed of two parts of sugar and protein connected by covalent bonds. Plant-derived glycoprotein performs various pharmacological activities in the body, such as anti-inflammatory (5), antihyperglycemia (6), antiproliferation (7), antioxidant (8), antiallergy (9), and immunomodulatory. Among them, immunomodulatory activity was the most widely reported. Niu et al. (10) demonstrated the immunomodulatory activity of Chinese yam glycoprotein by evaluating its effect on the production of TNF-α, interleukin-6 (IL-6), and NO in peritoneal macrophages. Nowadays, studies on the relationship between the chemical structure and bioactivity of glycoproteins have attracted great attention. In a previous study, the hypoglycemic activity of glycoproteins isolated from pea was found to be related to molecular weight (6).

As one of the most consumed legumes worldwide, chickpea (*Cicer arietinum* L.) represents nearly 20% of global legume production (11). Chickpea has a long history of planting and application in China due to its pharmacology activities, especially in Uygur traditional Chinese medicine (12). Accumulated studies showed that chickpea is rich in functional phytochemicals, such as isoflavones and peptides, which have physiological effects such as antidiabetic (9) and antiproliferative activities (13). The water-extracted polysaccharide in chickpea was claimed to have many biological activities, such as antioxidant activity (14) and angiotensin I converting enzyme (ACE-I) inhibitory activity (15). In addition, three polysaccharides were obtained under neutral conditions and performed a strong immunomodulatory activity in immunodeficient mice (16). However, to the best of our knowledge, there is a rare study that focuses on the chemical structure and immunomodulatory activity of chickpea glycoprotein. Therefore, in this study, a glycoprotein fraction was isolated from chickpea [crude chickpea glycoprotein-1 (CAG-1)] and purified with ion-exchange chromatography and gel-filtration chromatography, and the chemical structure was characterized by multiple methods. Then, the *in vitro* immunomodulatory activity was evaluated.

## Materials and methods

### Materials and reagents

Chickpea seeds (Xinying No. 3) were obtained from the Xinjiang Academy of Agricultural Sciences. DEAE-Sepharose Fast Flow and Superdex-200 were obtained from GE Healthcare Bio-Sciences Co. (Piscataway, NJ, USA). Griess reagent, arabinose (Ara), rhamnose (Rha), xylose (Xyl), galacturonic acid (GalA), galactose (Gal), glucose (Glc), glucuronic acid (GlcA), amino acid standard solution (AAS18), and 3-(4,5-dimethylthiazol-2-yl)-2,5-diphenyltetrazolium bromide (MTT) were purchased from Sigma-Aldrich (St. Louis, Miss., USA). RPMI-1640 media, Dulbecco's modified Eagle's medium (DMEM), lipopolysaccharide (LPS), phosphate-buffered saline (PBS), and fetal bovine serum (FBS) were obtained from Gibco BRL Life Technologies (Grand Island, NY, USA). The RAW 264.7 murine macrophage cell line was purchased from the Cell Resources Centre of the Chinese Academy of Sciences (Shanghai, China). OptEIA enzyme-linked immunosorbent assay (ELISA) kits for detecting tumor necrosis factor-α (TNF-α), monocyte chemotactic protein 1 (MCP-1), and IL-6 were purchased from BD Biosciences (San Diego, CA, USA). All other chemicals and solvents used were of analytical grade unless otherwise specified.

### Extraction of CAG

The CAG was extracted according to our previous method (3) with some modifications. Chickpea seed powder was extracted with 95% ethanol (1:10 w/v) for 24 h and then with alkaline water (1:20 w/v, pH 9.0) two times for 4 h (50°C). Non-extractable solid was removed by centrifugation (4,000 g, 15 min). The supernatant was precipitated with 95% ice-cold ethanol (1:4, v/v) overnight. The precipitate was collected and redissolved in distilled water. The Sevage method was used to remove free protein. The supernatant was collected and precipitated with 95% ice-cold ethanol (1:4, v/v) overnight, again, to obtain the CAG.

### Purification of glycoproteins

The CAG was dissolved in distilled water with centrifugation (10,000 g, 10 min), and then, the supernatant was loaded onto an ÄKTA explorer 100 purification system with a DEAE-Sepharose Fast Flow column (2.6 cm^2^ × 100 cm), which was first washed with distilled water and the bound polysaccharides were eluted with a 0–2.0 M NaCl gradient at a flow rate of 4 ml/min. Two elution peaks were obtained in the profile, and only the first fraction was studied due to its high yield. After that, the first fractions were dialyzed, lyophilized, and further purified with a Superdex-200 column. A high-performance liquid chromatography system (LC-20AT, Shimadzu, Japan) with a Shimadzu RID-10A refractive index detector (Kyoto, Japan) was used to monitor the elution. One glycoprotein, named CAG-1, was collected, dialyzed, and lyophilized. Polysaccharide content and protein content were determined, respectively, by the Kjeldah method and the phenol–sulfuric acid reaction method.

### Determination of molecular weight

The molecular weight of CAG-1 was measured using a high performance size exclusion chromatography system coupled with a multi-angle laser light scattering and refractive index (HPSEC-MALLS-RID) detector, which consisted of a pump (LC-20AD, Shimadzu, Kyoto, Japan), an HPSEC column (SB-805HQ, Shodex, Kyoto, Japan), a MALLS detector (DAWN HELEOS-II, Wyatt Technology, Santa Barbara, CA, USA), and an RI detector (Optilab Rex, Wyatt Technology, Santa Barbara, CA, USA). Samples were filtered through a 0.45-μm pore membrane before injection (200 μl) and eluted with 0.1 M NaCl (0.5 ml/min). The column temperature was maintained at 40°C.

### Determination of monosaccharide composition

The method of determining the composition of monosaccharides was carried out in a previous study (17). Approximately 2 mg of CAG-1 was mixed with 1 ml of trifluoroacetic acid (TFA) (2 M) to hydrolyze (120°C, 90 min) and evaporated to dryness on a rotary evaporator. Approximately 2 ml of double-distilled water and 100 mg of sodium borohydride were added to reduce the residue, and glacial acetic acid to neutralize it, and then, it was evaporated by rotary steam and oven dried at 110°C. Approximately 1 ml of acetic anhydride was added and heated at 100°C for 1 h and 3 ml of toluene was added and evaporated to dryness after cooling, repeated four to five times to remove excess acetic anhydride. The acetylated product was dissolved in 3 ml of chloroform and transferred to a separatory funnel. After adding distilled water and shaking completely, the upper aqueous solution was removed for five times. The chloroform layer was dried with an appropriate amount of anhydrous sodium sulfate to a constant volume of 10 ml. The gas chromatography-mass spectrometer (GC-MS) (Shimadzu, GCMS-QP 2010, Kyoto, Japan) was used for sample analysis of the acetylation product.

### Determination of amino acid composition

Gas chromatography-mass spectrometry was used to determine the amino acid composition of samples (18). Briefly, the sample was mixed with 6 M HCl in a hydrolysis tube and hydrolyzed at 100°C for 12 h. The solvent was removed from HCl by rotary evaporation, n-butanol was added, incubated at 100°C for 1 h, and then rotated to dryness. Acetylate trifluoroacetic anhydride was mixed with the sample at 50°C for 10 min. The standards used the same derivation method. The reaction was stopped with water and extracted with dichloromethane (CH_2_Cl_2_). In addition, the conditions of GC-MS were listed as follows: the chromatographic column was 30 m × 0.25 mm × 0.25 μm (RXI-5 SIL MS, Shimadzu, Tokyo, Japan); the temperature program conditions were an initial temperature of 60°C, heating at 4°C/min up to 280°C/min and maintained for 5 min, the inlet temperature was 250°C, the detector temperature was 250°C/min, the carrier gas was helium, and the flow rate was 1 ml/min.

### Analysis of Fourier transform infrared spectroscopy spectra

Functional groups of CAG-1 were analyzed by Fourier transform infrared (FT-IR) spectroscopy (Bruker, Rheinstetten, Germany). Approximately 2 mg of the sample was mixed with 200 mg of KBr and completely ground. The KBr powder was considered blank. They were placed in the pellets for scanning and recording in the range of 4,000–400 cm^−1^.

### Methylation analysis

Methylation analysis was carried out according to a previously described method (19). Briefly, 10 mg of CAG-1 was added with 100 μl of dimethyl sulfoxide and 2 mg of NaOH, sealed and ultrasonically dissolved. Approximately 1 ml of methyl iodide (CH_3_I) was added and reacted at 30°C for 60 min. Finally, 2 ml of ultrapure water was added to the above mixture to terminate the methylation reaction.

The methylated sample was mixed with 1 ml of 2 M TFA and hydrolyzed for 90 min and subsequently evaporated by a rotary evaporator. The residue was reduced by adding 2 ml of double-distilled water and 60 mg of sodium borohydride for 8 h, followed by adding glacial acetic acid for neutralization and rotary steaming at 101°C. Approximately 1 ml of acetic anhydride was added into the solution and reacted at 100°C for 1 h and cooled. Then, 3 ml of toluene was added to remove excess acetic anhydride for five times. The acetylated sample was dissolved with 3 ml of CH_2_Cl_2_ and transferred to a separatory funnel. After adding a small amount of distilled water and shaking sufficiently, the upper aqueous solution was removed four times. The CH_2_Cl_2_ layer was dried with an appropriate amount of anhydrous sodium sulfate, fixed to a volume of 10 ml, and placed in a liquid phase vial. The Shimadzu GCMS-QP 201 GC-MS was used to determine the acetylated product samples.

### ^1^H and ^13^C nuclear magnetic resonance analysis

The purified CAG-1 sample (20 mg) was dissolved in a D_2_O solution at 20°C. Bruker Avance 600 and Bruker Avance 500 nuclear magnetic resonance (NMR) spectrometers (Bruker, Ettlingen Germany) were used (operating frequencies of ^1^H: 600.1 and 499.8 MHz, operating frequencies of ^13^C: 150.9 and 125.7 MHz). ^1^H and ^13^C spectra, Dept135, HSQC, HHCOSY, and HMBC spectra, and CWP and CWP-0.2 spectra were recorded at 30 MHz with an MBC spectrometer (Bruker, Rheinstetten, Germany).

### Immunostimulatory activity analysis

The evaluation of the immunostimulatory activity was performed by the previous method in the laboratory (3). RAW 264.7 cells in the logarithmic growth phase were washed two times with PBS, digested with trypsin, and then centrifuged (4°C, 1,000 rpm, 4 min). After resuspending the cell, the cell density was adjusted to 2.5 × 10^6^ cells/ml in RPMI-1640 complete medium. Then, 100 μl of cells were added to a 96-well plate, placed in a CO_2_ incubator (5% CO_2_, 37°C, saturated humidity), cultured for 24 h, and the medium was subsequently discarded. Approximately 100 μl of cells and 100 μl of samples (20, 40, 60, 80, and 100 μg/ml) were added in a 96-well plate, and the LPS solution was used as a control group with the concentration of 1 μg/ml. Roughly 100 μl of culture medium was considered as a blank group. Among them, they were placed in a CO_2_ incubator (5% CO_2_, 37°C, saturated humidity) for 24 h, and 50 μl of each of the supernatants of each group was taken after 24 h adding 50 μl of Griess reagent, then reacted at room temperature for 15 min. After removing the bubbles, the absorbance at 540 nm was measured, and the amount of immune mediator (NO) in the supernatant was calculated. The production of TNF-α, MCP-1, and IL-6 cytokines was measured by ELISA kits according to the instructions (Becton, Dickinson and Company, NJ, USA).

Cytoxiocity of CAG-1 on RAW 264.7 cell was evaluated according to the previous method with some modifications (20). Cells in the logarithmic growth phase were washed two times with PBS, digested with trypsin, and then centrifuged (4°C, 1,000 rpm, 4 min). After resuspending the cell, the cell density was adjusted to 2.5 × 10^6^ cells/ml in RPMI-1640 complete medium. Then, 100 μl of cells were added to a 96-well plate, placed in a CO_2_ incubator (5% CO_2_, 37°C, saturated humidity), and cultured for 24 h, and the medium was subsequently discarded. Approximately 100 μl of RPMI-1640 complete medium contained in samples (20, 40, 60, 80, and 100 μg /ml) was added in each well after washing two times with PBS cultured for 24 h. Approximately 10 μl of MTT reagent (5 mg/ml) was added and placed in a CO_2_ incubator and was continued to culture for 4 h. Dimethyl sulfoxide was added to each well after washing two times with PBS and incubated for 1 h. The absorbance of each well solution was measured at a wavelength of 570 nm, and the cell viability was calculated based on the absorbance.


(1) $$\begin{array}{l} Cell\;survival\;rate\;\left(\%\right)\;=\;(absorbance\;of\;sample\\ \;/\;absorbance\;of\;control\;group)\;\times \;100 \end{array}$$

### Statistical analysis

Data were presented as mean ± standard deviation (SD). Statistical analysis was performed by GraphPad Prism 8.0.2 (GraphPad Software, San Diego, CA, USA) Statistical significance of differences was considered statistically significant at a *p* < 0.05 and assessed by a one-way analysis of variance (ANOVA) test.

## Results and discussion

### Purification of CAG

**Figure 1:**
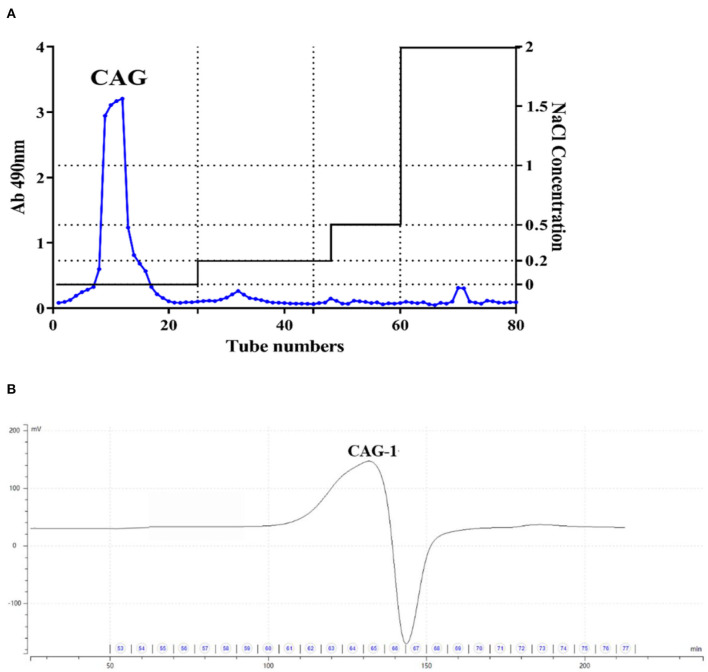
Purification of chickpea glycoproteins. **(A)** Crude chickpea glycoprotein (CAG) was passed from the DEAE-Sepharose column; **(B)** A purified fraction from the Superdex-200 column.

Chickpea seed powder was extracted under alkali conditions to obtain alkali-extracted crude chickpea glycoprotein. CAG was further purified with a DEAE-Sepharose column with a yield of 2.15% (Figure 1A). Then, it was filtered again through a Superdex-200 column, and one glycoprotein (CAG-1) was obtained (Figure 1B) with a yield of 16.8%. The polysaccharide content and protein content of CAG-1 were determined as 57.12 and 35.41% (Table 1), respectively. Glycoproteins contain more proteins than polysaccharides, and Qin et al. (6) reported that glycoproteins isolated from legumes (peas) with hot water contained 55.98–85.93% protein and 12.0–41.94% of polysaccharide. However, the extraction condition significantly influences the composition of the extracted products. Chen et al. (21) found that alkali-soluble polysaccharide/protein conjugates contained more neutral sugar and uronic acid than protein. In previous studies, the molecular weight of plant-derived glycoproteins was reported to be 14.4–897.4 kDa (6, 22). In the present study, the molecular weight of CAG-1 was determined to be 8,106 Da (Table 1), which is lower than the previous results. The slight differences with our results could also be due to differences in extraction methods.

**Table 1:** The polysaccharide content, protein content, and molecular weight of crude chickpea glycoprotein-1 (CAG-1).

**Sample ID**	**Polysaccharide**	**Protein**	**Mw**
	**content (%)**	**content (%)**	**(Da)**
CAG-1	57.1 ± 2.3	35.4 ± 3.2	8,106

### FT-IR spectra analysis

**Figure 2:**
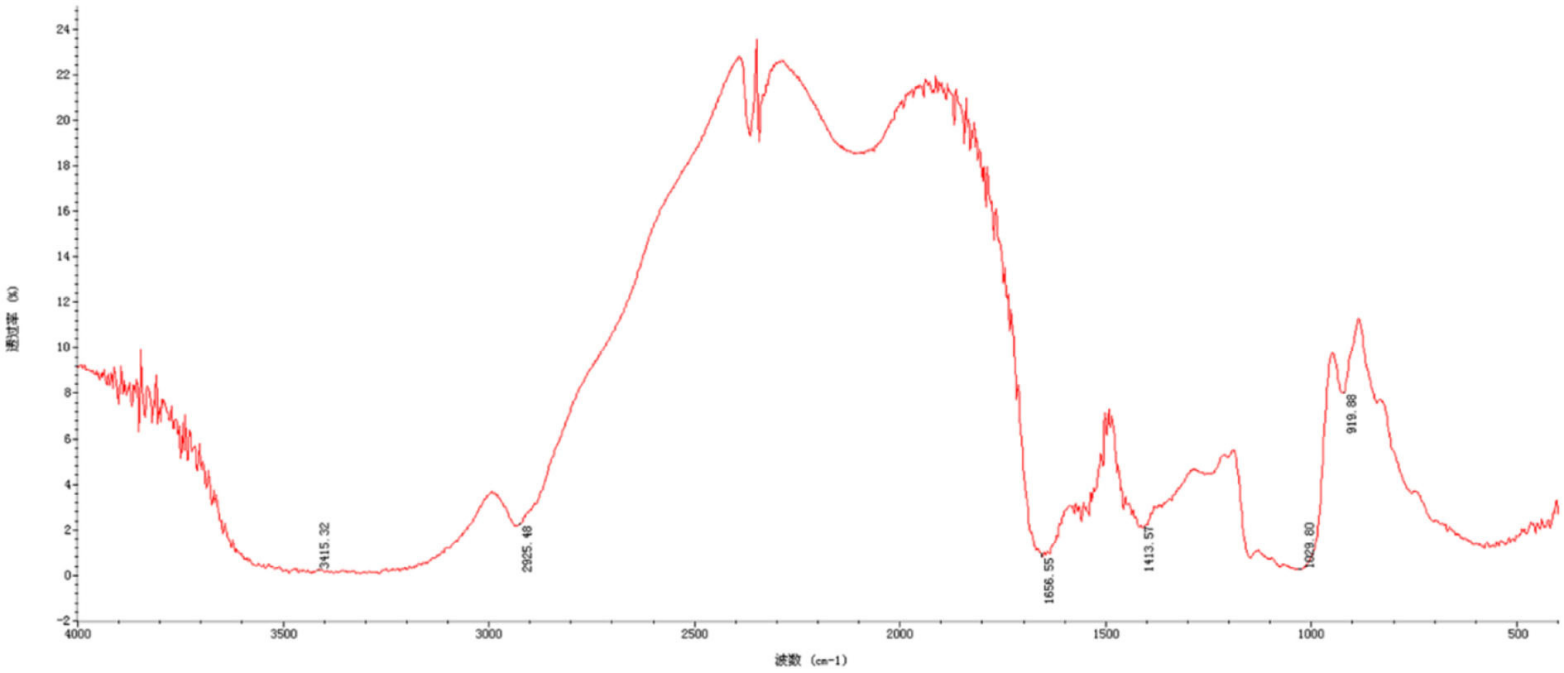
Fourier-transform infrared (FT-IR) spectrum of CAG-1.

Fourier transform infrared spectroscopy spectra indicated chemical functional groups in the samples. As seen in Figure 2, the results of CAG-1 indicated that the absorption peak at 3,415 and 2,925 cm^−1^ were the O-H stretching vibration and the C-H stretching vibration, whose absorption peak at 3,415 was the characteristic peak of sugars (23). Meanwhile, the absorption peak at 1,656 cm^−1^ was induced by C=O asymmetric stretching vibration (6, 24). In the range from 1,420 to 1,200 cm^−1^, the absorption peaks were caused by the variable-angle vibration of C-H (6). The main absorption peaks in this region were 1,413 and 1,243 cm^−1^. In addition, the absorption peak at 1,200–1,000 cm^−1^ was a wide peak, which was mainly caused by C-O stretching vibrations, namely C-O-H and C-O-C (6). The asymmetric ring stretching vibration was performed at absorption peaks of 919 cm^−1^.

### Monosaccharide composition and glycosidic linkage analysis

**Figure 3:**
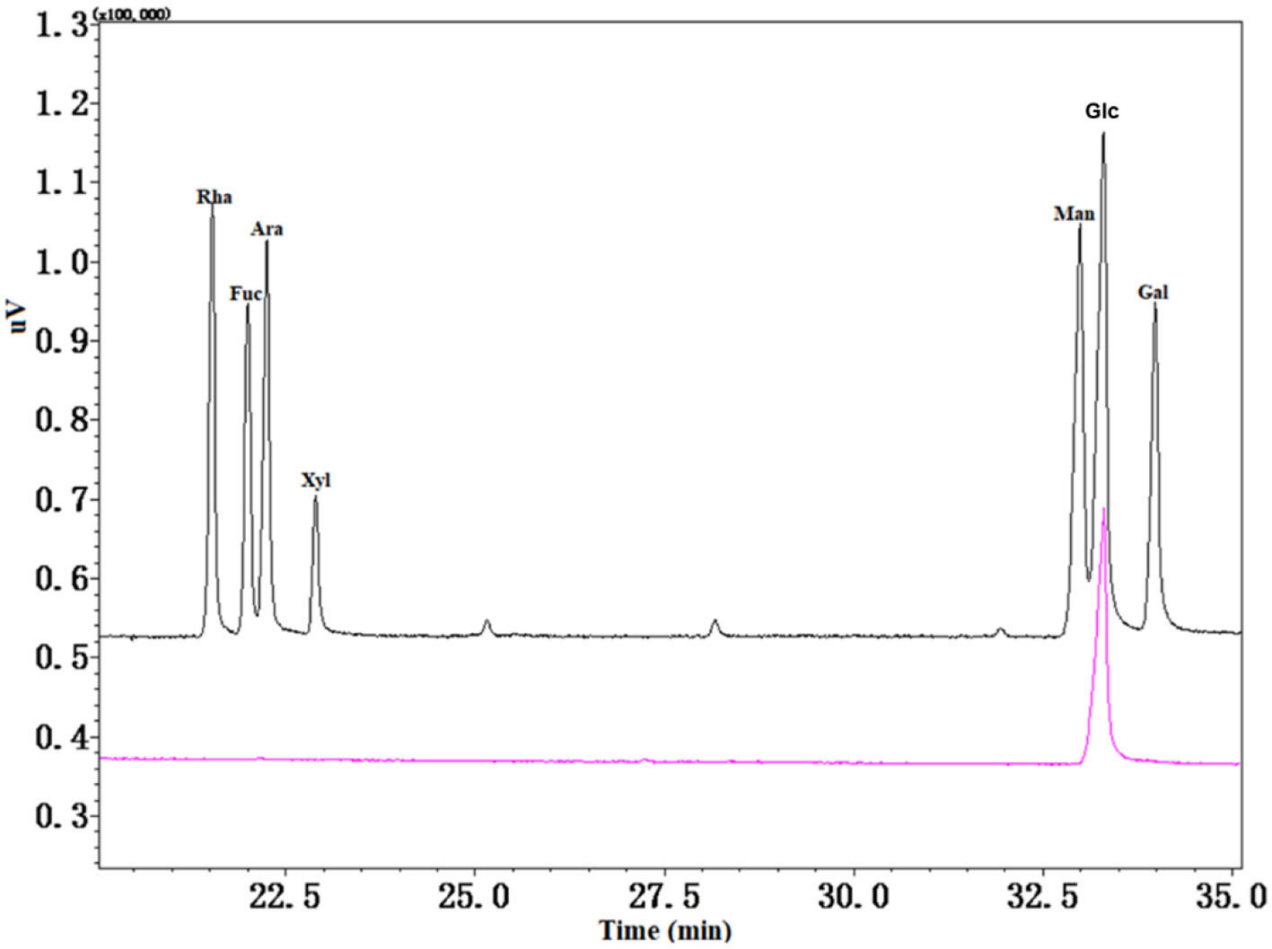
Gas chromatography-mass spectrometer (GC-MS) spectra of monosaccharide composition in CAG-1.

Seven monosaccharides were used as a standard to measure the monosaccharide composition of CAG-1 by GC-MS analysis. As seen in Figure 3, only Glc was found. A previous study showed that arabinan (Ara) was rich in chickpea hull (25); however, Ara was not detected in CAG-1, which may be in part because CAG-1 originated from chickpea seeds rather than chickpea hulls. On the contrary, the alkali solution was beneficial for the dissolution of Glc. Through methylation analysis, it has been found that CAG-1 has five kinds of glycosidic bonds. Among them, → 4)-Glc*p*-(1 → and → 4,6)-Glc*p*-(1 → were the main glycosidic bonds detected (Table 2).

**Table 2:** Glycosidic bonds and types of linkage in CAG-1.

**RT (min)**	**Glycosidic bonds**	**Mass fragments (m/z)**	**Molar ratios**	**Type of linkage**
16.239	2,3,4,6-Me_4_-Glc*p*	43,71,87,101,117,129,145,161,205	0.14	Glc*p*-(1 →
21.378	2,3,6-Me_3_-Glc*p*	43,87,99,101,113,117,129,131,161,173,233	0.53	→ 4)-Glc*p*-(1 →
24.723	2,6-Me_2_-Glc*p*	43,87,97,117,159,185	0.10	→ 3,4)-Glc*p*-(1 →
27.242	2,3-Me_2_-Glc*p*	43,71,85,87,99,101,117,127,159,161,201	0.20	→ 4,6)-Glc*p*-(1 →
30.09	2-Me_1_-Glc*p*	43,58,87,97,117,139	0.03	→ 3,4,6)-Glc*p*-(1 →

### Amino acid composition analysis

**Figure 4:**
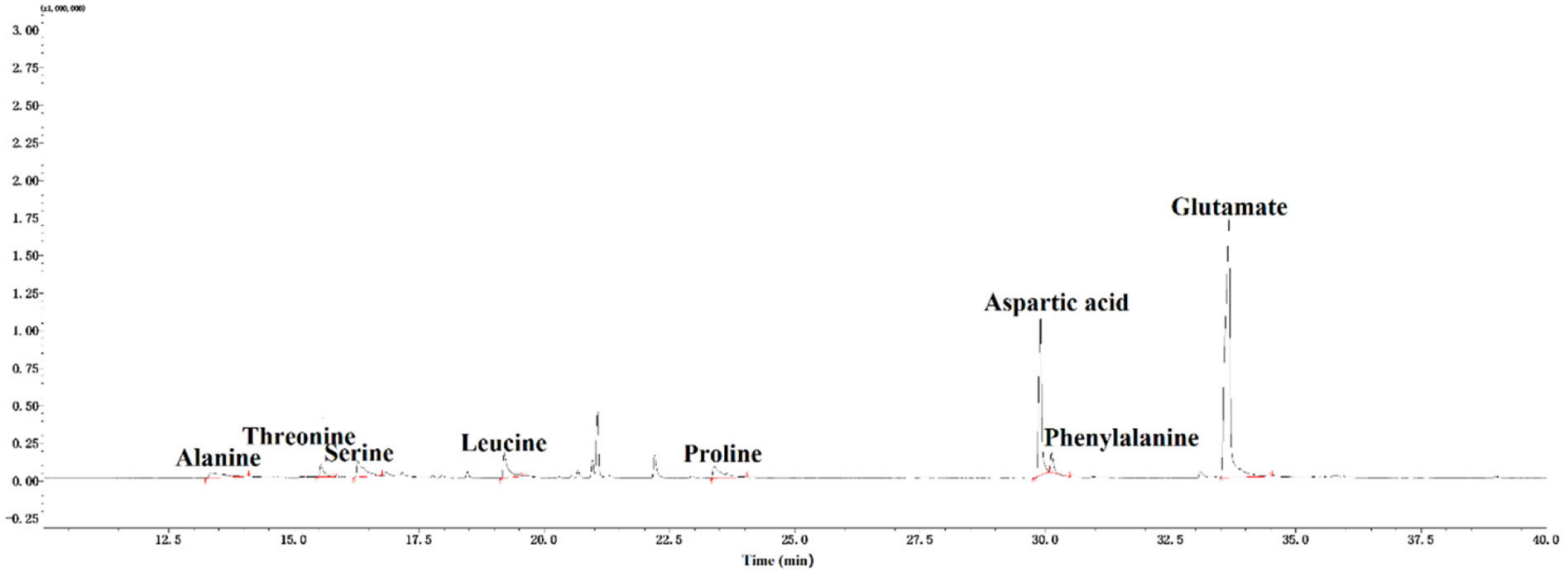
GC-MS spectra of amino acids in CAG-1.

A total of eight amino acids, which included alanine (Ala), threonine (Thr), serine (Ser), leucine (Leu), proline (Pro), aspartic (Asp), phenylalanine (Phe), and glutamic (Glu), were found in CAG-1 (Figure 4). Based on the peak area, the relative amino acid content was calculated. As seen in Table 3, the content of Glu and Asp was higher than that of others. The bulky side chains of Glu affected the activity of acetylcholinesterase, which was important in the treatment of Alzheimer's disease (26). In addition, Ala, Thr, and Ser existed in CAG-1. More information was obtained by analytical NMR.

**Table 3:** Amino acids in CAG-1.

**RT (min)**	**Type**	**Content (g/100 g)**
13.348	Ala	1.1
15.537	Thr	0.7
16.27	Ser	1.8
19.195	Leu	1.8
23.385	Pro	1.6
29.901	Asp	7.1
30.124	Phe	0.8
33.664	Glu	20.4

### NMR spectroscopy analysis

**Figure 5:**
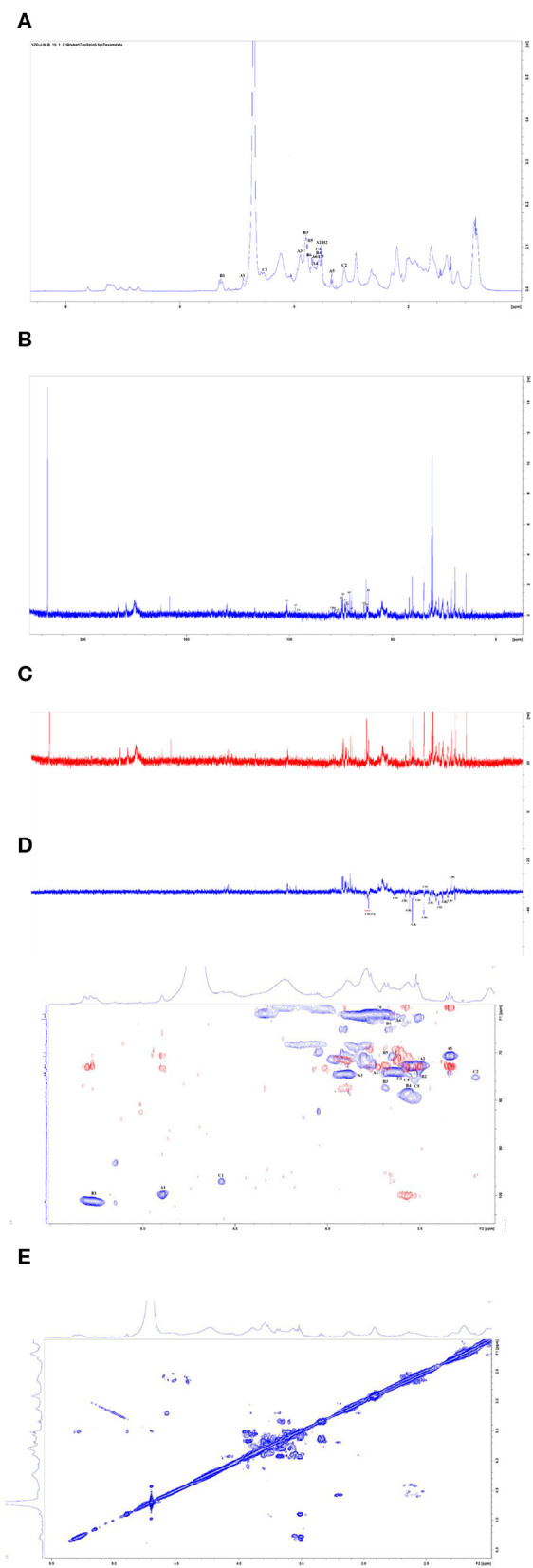
Nuclear magnetic resonance (NMR) spectra of CAG-1 in D_2_O. **(A)** ^1^H spectra; **(B)** ^13^C spectra; **(C)** DEPT_135_; **(D)** HSQC (in blue) + HMBC spectra (in red); and **(E)** COSY spectra.

The ^1^H NMR spectrum, ^13^C NMR spectrum, DEPT135 one-dimensional spectrum, and two-dimensional spectrum were measured with an NMR instrument, and the results of CAG-1 are illustrated in Figure 5. These signal peaks were distributed mainly in the 1–8 ppm area according to the ^1^H NMR spectrum (Figure 5A). In addition, the 0–3.2, 3.2–5.2, and 6.5–8 ppm regions were mainly attributed to aliphatic alkane hydrogen signal peaks, polysaccharide hydrogen signal peaks, and the aromatic hydrogen signal peaks, respectively (Figure 5A). According to the ^13^C NMR spectrum, 130.1 and 130.5 ppm were attributed to the aromatic benzene ring peaks and 158.1 and 162.2 ppm to the amide bond carbon. Moreover, 174.2, 174.9, 179.1, and 182.7 ppm were the amino acid carboxyl group, and 97.1 and 101.1 ppm were the dextran anomeric hydrogen signal peaks (Figure 5B). According to the ^13^C NMR and DEPT135 spectra, methylene signal peaks were mainly 23.50, 25.92, 27.75, 29.01, 32.54, 34.92, 35.04, 39.96, 40.66, 41.98, 43.98, 49.37, 61.73, 62.56, and 63.02 ppm, of which 61.73, 62.56, and 63.02 ppm were the sugar C_6_ signal peaks (Figure 5C). Others were the methylene signal peaks in the polypeptide, while 14.51, 19.93, and 21.61 ppm were methyl signals. The results of methylation demonstrated that the glycosidic bond of the polysaccharide part of the glycoprotein was mainly composed of Glc*p*-(1 → 4)-Glc*p*-(1 → , so the polysaccharide part of the glycoprotein was mainly 1,4-linked dextran sugar.

According to the HSQC spectrum, δ101.23 was observed, which was the anomeric carbon signal, and the corresponding anomeric hydrogen signal in the HSQC spectrum was δ5.29 (Figure 5D). The HH-COSY, used to mark the signal of H_1−2_, H_2−3−_, and H_3−4_, indicated that H_1_, H_2_, H_3_, and H_4_ were δ5.29, δ3.5, δ3.89, and δ3.57, respectively (Figure 5E). The corresponding chemical shifts of C_1_–C_4_ were δ101.23, δ72.75, δ74.66, and δ78.6. The corresponding C_5_ was δ 72.45, and its chemical shift of C_6_ was δ61.73. Therefore, the signal attributed to the glycosidic bond → 4)-α-Glc*p*-(1 → . It was observed that the anomeric carbon signal was δ99.71, and the corresponding anomeric hydrogen signal in the HSQC spectrum was δ4.89 (Figure 5D). When the HH-COSY appeared, the signal of H_1−2_ was 4.89/3.50, and the signal of H_2−3_ was 3.50/3.92. Meanwhile, the signal of H_3−4_ was 3.92/3.66 (Figure 5E). It was deduced that H_1_, H_2_, H_3_, and H_4_ were δ4.89, δ3.5, δ3.92, and δ3.66, respectively. The corresponding chemical shifts from C_1_-C_4_ were δ99.71, δ73.052, δ74.88, and δ73.88. The corresponding C_5_ was 70.50, and the chemical shift of C_6_ was δ63.02. Therefore, the signal should be attributed to the glycosidic bond α-Glc*p*-(1 → , and the correlation peaks are presented in Table 4.

**Table 4:** ^1^H and ^13^C nuclear magnetic resonance (NMR) chemical shifts in CAG-1 (ppm).

**Glycosyl residues**	**H1a,b**	**H2**	**H3**	**H4**	**H5**	**H6a**	**H6b**
	**C1**	**C2**	**C3**	**C4**	**C5**	**C6**	
Residue A	4.89	3.5	3.92	3.66	3.35	3.68	
α-d-Glc*p*-(1 →	99.71	73.052	74.88	73.88	70.5	63.02	
Residue B	5.29	3.5	3.89	3.57	3.76	3.73	3.76
→ 4)-α-d-Glc*p*-(1 →	101.23	72.75	74.66	78.6	72.45	61.73	
Residue C	4.57	3.2	3.68	3.58	3.53	3.77	
→ 4)-β-d-Glc*p*	97.2	75.07	77.58	78.75	75.9	61.99	

HMBC analysis showed that the anomeric hydrogen of the polysaccharide has a correlation peak with its own C_4_, indicating the existence of → 4)-α-d-Glc*p*-(1 → 4)-α-d-Glc*p*-(1 → glycosidic bond. The anomeric carbon of α-d-Glc*p*-(1 → and its → 4)-α-d-Glc*p*-(1 → H4) have correlation peaks, indicating the presence of α-d-Glc*p*-(1 → 4)-α-d-Glc*p*-(1 → 4)-α-d-Glc*p*-(1 → . In addition, the protein component of the glycoprotein was mainly connected by Asp acid and Glu acid and contains some Ala, Thr, Ser, Leu, Pro, and Phe. As the sample was extracted with alkaline water, similar to the β-elimination reaction, the sugar chains in the glycoprotein were not hydrolyzed under alkaline conditions, indicating that the polysaccharide had an N-glycosidic bond. The sugar chain was linked to the protein chain by Asp acid.

### Immunomodulatory activity

**Figure 6:**
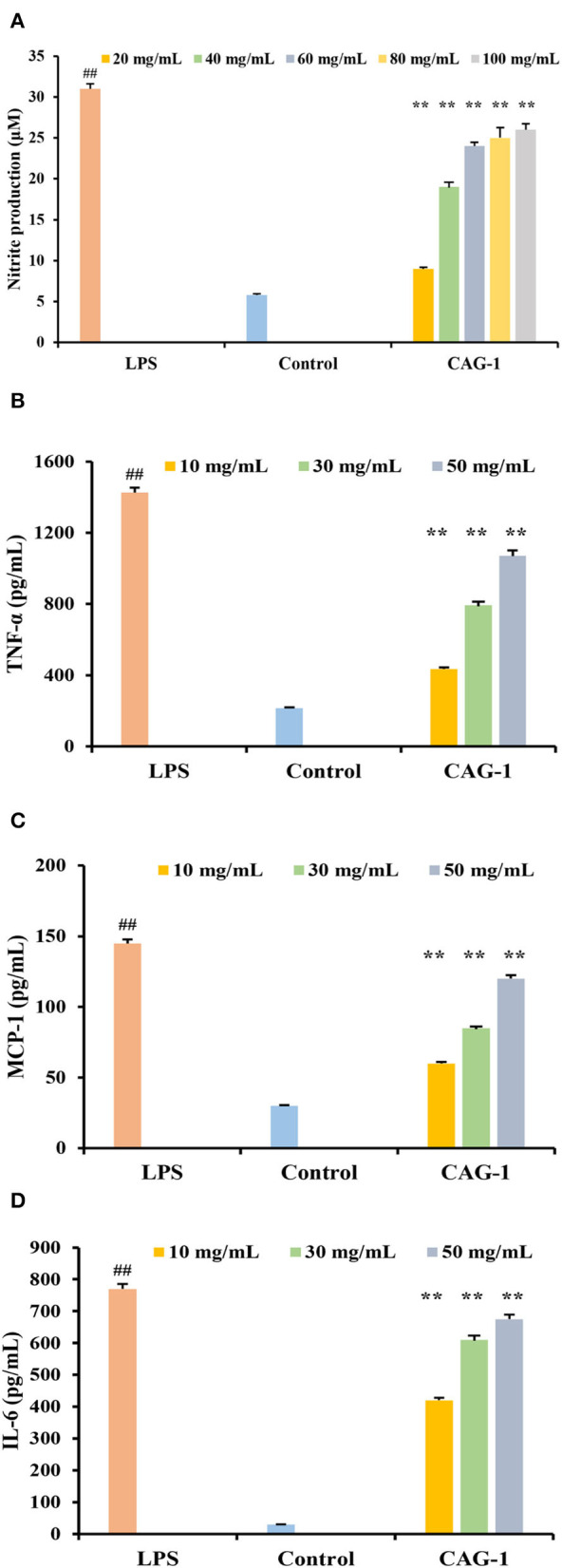
Effects of CAG-1 on the production of nitric oxide (NO), tumor necrosis factor-α (TNF-α), monocyte chemotactic protein 1 (MCP-1), and interleukin-6 (IL-6) in macrophages RAW 264.7 cells. **(A)** NO; **(B)** TNF-α; **(C)** MCP-1; and **(D)** IL-6. Values are mean ± standard deviation (SD) (*n* = 3). ^##^*p* < 0.01 compared to control and **p < 0.01 compared to lipopolysaccharide (LPS).

With an increase in sample concentration, the survival rate of RAW264.7 macrophages did not change significantly (*p* < 0.05) (data not shown). Immune activity was evaluated by cell viability, cellular NO release, TNF-α, MCP-1, and IL-6 (Figure 6). As an important signaling molecule, NO regulates a diverse range of physiological processes in many tissues. LPS caused cells to swell by increasing NO release, so the LPS group was considered a positive control (10). NO production in the control and LPS groups was 5.02 and 32.07 μM, respectively. At the experimental concentration (20–100 μg/ml), the production of NO was associated with the concentration of CAG-1. The production of NO in CAG-1 was 8.07 μM at 20 μg/ml, while it increased to 24.53 μM at 100 μg/ml, indicating its strong effect on macrophage activation. Associated with proinflammatory properties, TNF-α plays a key role in innate and adaptive immunity, especially in host defense mechanisms that terminate intracellular bacteria (27). Accumulating studies uncovered that TNF-α also plays important immunoregulatory roles and is directly associated with the maintenance of immune homeostasis (28). This indicates that the net effect of TNF-α is balanced between its immunosuppressive and proinflammatory functions and is decided by the cellular microenvironment and differs (29). In this study, TNF-α, MCP-1, and IL-6 were also significantly increased in the LPS group as the inflammation was caused by LPS (30, 31). In this study, the release of cytokines (TNF-α, MCP-1, and IL6) was significantly increased by 232.61, 162.09, and 86.52% compared to CAG-1 at 10 μg/ml, respectively. It indicated that CAG-1 moderated inflammation by LPS, which was also observed in glycoproteins isolated from *Cudrania tricuspidata* Bureau (32). In CAG-1 at 10 μg/ml, the TNF-α, MCP-1, and IL-6 levels were detected to be 428.30, 55.85, and 413.11 pg/ml, respectively.

## Conclusion

One alkali-soluble glycoprotein (CAG-1), with a molecular weight of 8,106 Da, was purified from chickpea seeds. Glc was found to be the main component of the polysaccharide part of CAG-1, and → 4)-Glc*p*-(1 → and → 4,6)-Glc*p*-(1 → were the main glycosidic bonds detected in CAG-1. A total of eight amino acids were detected in CAG-1; among them, Asp and Glu were the two most important amino acids. The results of NMR analysis indicated the presence of α-d-Glc*p*-(1 → 4)-α-d-Glc*p*-(1 → 4)-α-d-Glc*p*-(1 → . Moreover, the sugar chains in glycoproteins were not hydrolyzed under alkaline conditions, suggesting that the polysaccharide had an N-glycosidic bond, by which the sugar chain was linked to the protein chain by Asp acid. An immunological study showed that CAG-1 stimulated the production of NO, IL-6, TNF-α, and MCP-1 in RAW 264.7 macrophages in a dose-dependent manner. These results suggest that alkali-extracted glycoprotein of chickpea had immunomodulatory activities and could be beneficial for health.

## Data availability statement

The original contributions presented in the study are included in the article/supplementary material, further inquiries can be directed to the corresponding authors.

## Author contributions

ZS, SL, and ZW contributed to conception and design of the study. YW organized the database. NZ performed the statistical analysis. ZS wrote the first draft of the manuscript. SL, ZW, YW, and NZ wrote sections of the manuscript. All authors contributed to manuscript revision, read, and approved the submitted version.

## Funding

This work was supported by the National Key R&D Program of China (2021YFD1600100), the earmarked fund for the China Agriculture Research System (CARS-08-G21), the Key Laboratory of Grain Crop Genetic Resources Evaluation and Utilization, and the Science and Technology Research Projects of Chongqing Education Commission (KJQN202102705).

## Conflict of interest

The authors declare that the research was conducted in the absence of any commercial or financial relationships that could be construed as a potential conflict of interest.

## Publisher's note

All claims expressed in this article are solely those of the authors and do not necessarily represent those of their affiliated organizations, or those of the publisher, the editors and the reviewers. Any product that may be evaluated in this article, or claim that may be made by its manufacturer, is not guaranteed or endorsed by the publisher.
